# Dual-port distal gastrectomy for the early gastric cancer

**DOI:** 10.1007/s00464-014-3827-9

**Published:** 2014-08-27

**Authors:** Hiroyuki Kashiwagi, Kenta Kumagai, Eiji Monma, Mutsumi Nozue

**Affiliations:** Department of Surgery, Shonai Amarume Hospital, Shouyou 1-1-1, Shonai Town, Higashi-Tagawa, 999-7782 Japan

**Keywords:** Reduced port surgery, Laparoscopic distal gastrectomy, Multi-channel port

## Abstract

**Background:**

Although recent trends in laparoscopic procedures have been toward minimizing the number of incisions, four or five ports are normally required to complete laparoscopic gastrectomy because of the complexity of this procedure. Multi-channel ports, such as the SILS port (Covidien, JAPAN), are now available and are crucial for performing single-incision laparoscopic surgery (SILS) or reduced port surgery (RPS). We carried out reduced port distal gastrectomy (RPDG) using a dual-port method with a SILS port.

**Methods:**

Ten patients who were diagnosed as early stage gastric cancer were offered the RPDG. Mean age and body mass index (BMI) were 68.1 and 21.4, respectively. No distant metastasis or regional lymph node swelling was seen in any case. A 5-mm flexible scope (Olympus, JAPAN) and SILS port were used and a nylon ligature with a straight needle, instead of a surgical instrument, was available to raise the gastric wall.

**Results:**

The average operative time was 266.9 ± 38.3 min and blood loss was 37.8 ± 56.8 ml. Patients recovered well and experienced no complications after surgery. All patients could tolerate soft meals on the first day after surgery and the average hospital stay was 8.1 days. Past conventional LAG cases were evaluated to compare the short-term outcome and no difference was seen in the mean operative time or operative blood loss. The length of hospital stay after surgery was shorter for the RPDG group than the conventional operation group (*p* < 0.0001). Interestingly, the trend of serum CRP elevation after surgery was lower in the RPDG group than the conventional LAG group (*p* = 0.053).

**Conclusions:**

Although the benefits of RPS have not been established, this type of surgery may be expected to have some advantages. Cosmetic benefits and shorter hospital stays are clear advantages. Less invasiveness can be expected according to the trend of serum CRP elevation after RPDG.

Laparoscopic surgery is a modern operative technique that has brought a number of advantages to patients compared to conventional open procedures. These include reduced pain, shorter recovery time, reduced surgical site infection, and cosmetic benefits [1, 2]. Despite the rapid development of laparoscopic surgery in the last decade, laparoscopic gastrectomy (LAG) is still limited because of the complexity of the procedure [3, 4]. Although recent trends in laparoscopic procedures have been toward minimizing the number of incisions, four or five incisions (ports) or additional small incisions are normally required to complete this operation [5, 6]. These assistant ports are used for visualization and/or to counter traction against dissected tissues during LAG.

Multi-channel ports, which enable insertion of multiple instruments via a single incision, are now available worldwide. Recently, single-incision laparoscopic surgery (SILS) or reduced port surgery (RPS), using a multi-channel port to achieve less invasive surgery has been reported [7, 8]. A number of instruments are available to support this procedure. The SILS port (Covidien, JAPAN) is such a valuable instrument and enables insertion of three instruments through a single incision in the umbilicus. Some technical problems associated with SILS have been reported: restriction of the working field and interference of surgical instruments in the visceral space [9]. The principle of laparoscopic surgery requires triangulation in terms of visualization using a camera and maneuvers with both hands. The SILS technique essentially requires a single dimension of surgical instruments; however, an additional port and other lifting device can overcome this problem. We report our experience with reduced port distal gastrectomy (RPDG) with dual ports and demonstrate its safety and efficacy and compare that method to conventional LAG.

## Materials and methods

Ten patients were enrolled in this study from December 2010 to December 2012 and all were operated on by the same surgeon in one institution. The study population comprised four women and six men with a mean age of 68.1 years (range 52–87 years). Their BMI ranged from 17.8 to 23.5 kg/m^2^ (mean BMI 21.4 kg/m^2^). Four (40 %) of the patients were over 75 years old. Some patients had co-morbid diseases, such as diabetes, hypertension, cardiovascular disease, chronic obstructive pulmonary disease, chronic renal failure, and past history of cerebral infarction (Table 1). The grade of the Eastern Cooperative Oncology Group (ECOG) performance status [10] was used to evaluate the patients’ activity.

**Table 1:** Comparison of patient characteristics

	Dual port (*n* = 10)	Conventional (*n* = 9)	*p* value
Age (mean ± SD)	52–87 (68.1 ± 11.0)	55–81 (70.8 ± 8.0)	0.447
Male/female	6/4	5/4	–
Mean BMI (kg/m^2^)	21.4 ± 1.91	22.4 ± 2.16	0.211
Performance status			
0	6	5	0.964
1	1	2	0.466
2	3	2	0.701
Comorbid disease			
Diabetes mellitus	1	1	0.937
Hypertension	1	5	0.033
Cardiovascular	1	1	0.937
Respiratory	1	0	0.330
CRF	1	1	0.937
Cerebral infarction	1	0	0.330
Reconstruction			
Roux-en-Y	10	6	–
Billroth I	0	3	–
With small incision (less than 5 cm)	0	5	–

*BMI* body mass index, *CRF* chronic renal failure

All patients were diagnosed preoperatively as clinical stage T1 of early gastric cancer by endoscopic findings, biopsy specimens, and other graphical studies. The cases of contra-indication of endoscopic submucosal dissection (ESD) or additional treatment after ESD according to guidelines of the Japanese Gastric Cancer Association [11], were nominated for laparoscopic distal gastrectomy. The tumors located in the middle or lower body of the stomach were identified. To recognize the location of cancer lesions laparoscopically, in all patients the proximal sides of the lesions were stained with tattoos during preoperative endoscopy.

**Fig. 1:**
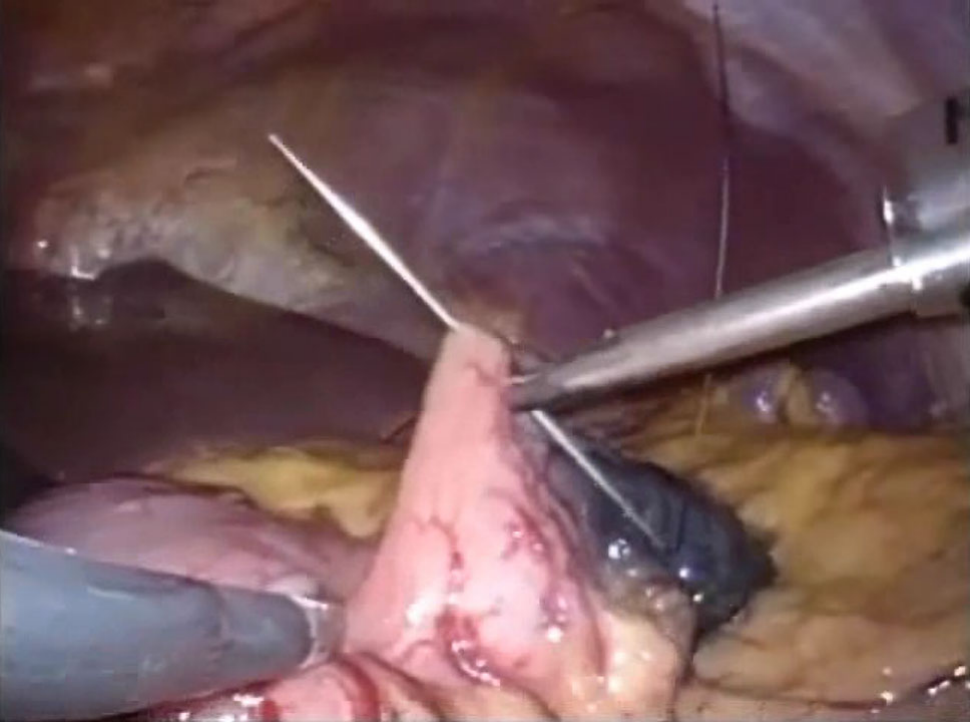
Surgical nylon with a straight needle was inserted into the visceral space and the anterior wall of the stomach was sutured

**Fig. 2:**
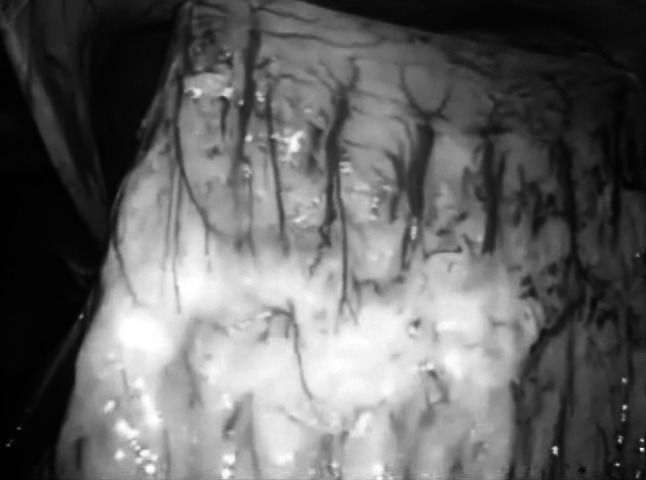
After lifting the gastric wall, the anatomical relationship between the gastric vessels and other neighboring organs was easily visualized

**Fig. 3:**
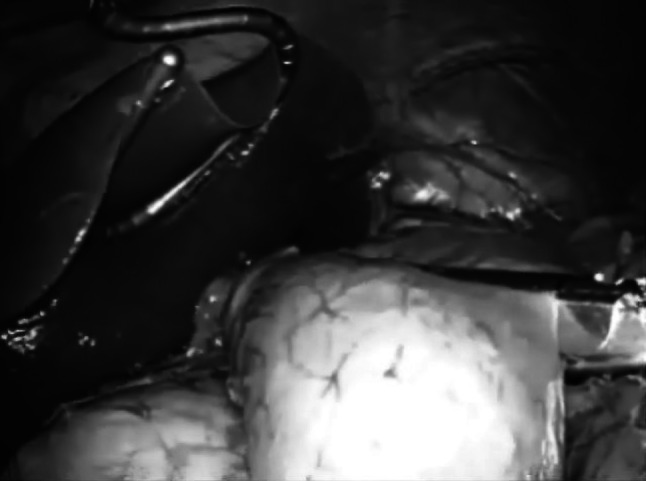
The proximal side of the tattoo staining was cut by endo-GIA to complete the distal gastrectomy

**Fig. 4:**
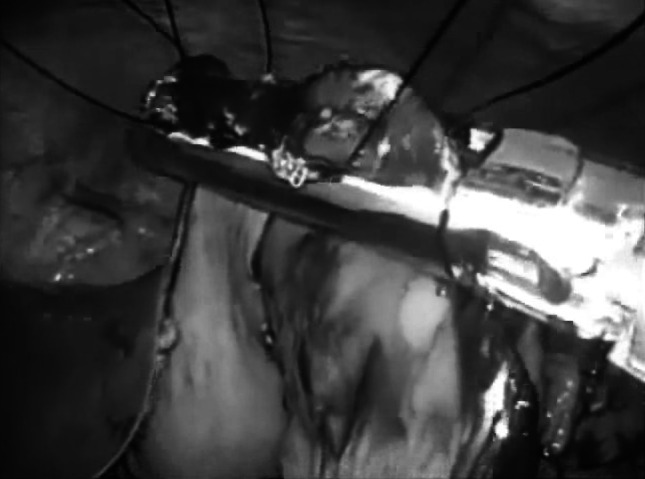
After completing the gastro-jejunostomy by endo-GIA, a surgical nylon ligature was used to close the suture hole

A SILS port and 5-mm flexible laparoscope (Olympus, JAPAN) were used in all cases. With the patient under general anesthesia in the lithotomy position, the SILS port was inserted into a 2.5-cm umbilical incision. After inspection of the visceral space, a second port was inserted into the left lower abdomen. To lift the gastric wall, a surgical nylon ligature with a straight needle was inserted into the abdomen without any ports and simply sutured to the anterior wall of the stomach (Fig. 1). After inserting two sutures into the stomach wall, the needles were removed from the abdomen. Then, the stomach wall was lifted and fixed temporarily to the abdominal wall to visualize the relationship between the gastric vessels and other neighboring organs (Fig. 2). The gastric vessels were identified easily after opening the brusa space. The distal side of the gastric vessels, such as the gastroepiploic arcade and right gastric artery, was dissected before cutting the duodenum by end-GIA (Covidien, JAPAN). The suturing nylon ligatures were released from the stomach after cutting the duodenum and the left gastric vessels were exposed and cut near their root. Distal gastrectomy was completed by endo-GIA, cutting along the proximal side of the tattoo injected preoperatively (Fig. 3). Roux-en-Y reconstruction was performed in all cases. After performing a gastro-jejunostomy by endo-GIA, surgical nylon was used to close the suture hole (Fig. 4). Jejuno-jejunostomy was performed outside the abdomen using the umbilical incision.

Data were analyzed using SPSS statistical software (SPSS Inc., Tokyo, JAPAN). Patient characteristics were compared using the Mann–Whitney *U* test. Quantitative variables were compared using Student’s *t* test and expressed as median ± SD. The Chi square statistic was used to test for differences in proportions. Probability (*p*) values were considered to be statistically significant at the <0.05 level.

## Results

All patients were transferred from the high care unit to the general unit the day after surgery and began to take soft meals. Table 2 shows the pathological outcome after RPDG. One case was diagnosed as a MALT lymphoma after pathological confirmation, including that by immunohistochemical studies. This case was diagnosed preoperatively with the suspicion of poorly differentiated adenocarcinoma. The distal and proximal surgical margins were sufficient to ensure safe resection in all cases. Almost all cases were diagnosed as clinical stage I gastric cancer according to the TNM classification and no patient had lymph node metastasis. Past cases operated on before starting RPDG were used for comparison with conventional LAG (Tables 1, 3). The patients’ characteristics were almost identical in the two groups. Five patients in the conventional LAG group had hypertension, which was treated with medication (*p* = 0.033). Three cases in the conventional LAG group received Billroth I reconstruction and 5 cases required an additional incision less than 5 cm.

**Table 2:** Pathological outcome after dual-port distal gastrectomy

Case no.	Location	Pre Dx	Post Dx	Size (mm)	PM (mm)	DM (mm)	*p* stage
1	M	Poor	MALT	15 × 11	35	70	I
2	M	Mod	Mod	32 × 20	25	60	Ia
3	L	Mod	Mod	35 × 35	40	23	IIa
4	L	Poor	Poor	15 × 8	100	12	Ib
5	L	Mod	Mod	45 × 34	22	50	Ia
6	ML	Mod	Mod/poor	65 × 25	80	20	Ia
7	L	Mod	Mod	12 × 12	60	47	Ia
8	M	Mod	Pap	28 × 12	25	99	Ia
9	L	Mod	Well	12 × 8	106	30	Ia
10	M	Mod	Well	25 × 15	28	72	Ia

*Pre Dx* pre-operative diagnosis, *Post Dx* post-operative diagnosis, *PM* proximal margin, *DM* distal margin

**Table 3:** Outcomes of surgical procedures

	Dual port (*n* = 10)	Conventional (*n* = 9)	*p* value
Operation time (min)	266.9 ± 38.3	255.3 ± 68.5	0.744
Intra-op. bleeding (ml)	37.8 ± 56.8	55.4 ± 57.1	0.129
Dissected lymph nodes (no.)	16.1 ± 8.9	14.9 ± 7.2	0.869
First flatus (days)	3.4 ± 1.1	3.5 ± 2.6	0.524
Times of pain drugs	3.1 ± 4.2	3.2 ± 3.2	0.901
Hospital stay after surgery	8.1 ± 1.5	17.3 ± 7.4	<0.0001
Complications			
Gastric stasis	0	2	–
Post-op. pneumonia	0	1	–
Anastomotic leakage	0	0	–
Wound problems	0	0	–
Conversion to open surgery	0	0	–
Mortality	0	0	–

There were no differences between the dual-port approach and the conventional multi-port method performed in 9 patients between 2008 and 2010 in terms of mean operative time (266.9 ± 38.3 vs. 255.3 ± 68.5 min, respectively), blood loss (37.8 ± 56.8 vs. 55.4 ± 57.1 ml, respectively), and retrieved lymph nodes (16.1 ± 8.9 vs. 14.9 ± 7.2, respectively). The postoperative hospital stay, however, was significantly longer in the conventional multi-port group (17.3 ± 7.4 vs. 8.1 ± 1.5 days, *p* < 0.0001). Three patients in that group experienced complications (one case of postoperative pneumonia and two cases of gastric stasis) compared to no complications in the dual-port group.

**Fig. 5:**
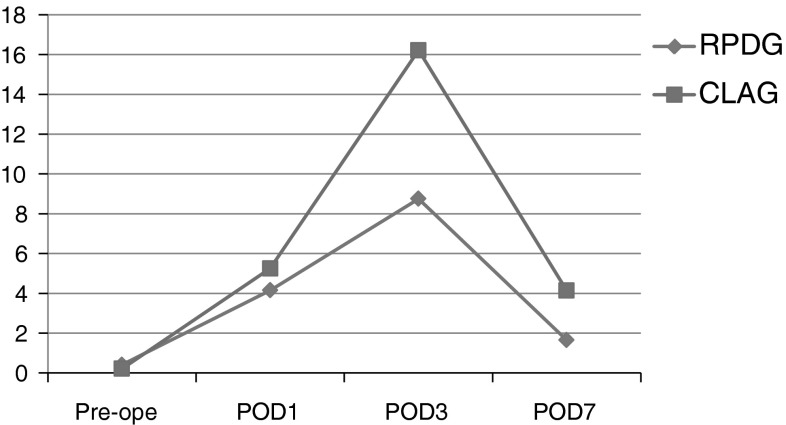
Trend of serum CRP values after surgery, indicating less invasiveness of RPDG than conventional LAG

For the comparison of invasiveness between the two groups, the mean value of serum C-reactive protein (CRP), a marker of inflammation, was evaluated at days 1, 3, and 7 after surgery. Because four patients in the RPDG group were discharged before day 7, the mean value of the remaining six was calculated for day 7. Pre-operative levels of CRP in the RPDG and conventional LAG groups were 0.28 and 0.21, respectively. The trend of mean CRP values in the RPDG group was below that of the conventional LAG group (Fig. 5). Interestingly, the peak CRP level seen at day 3, reflecting an acute reaction after surgery, differed between groups: 8.75 for RPDG and 16.22 for conventional LAG. Although this difference was not statistically significant, the p-value was relatively small (*p* = 0.053). Thereafter, the mean levels of CRP decreased gradually and the values for the RPLG and conventional LAG groups at day 7 were 1.65 and 4.14, respectively.

## Discussion

Recently, a trend in the refinement of laparoscopic procedures has been toward minimizing the number of incisions to reduce invasiveness. One such approach is SILS. SILS was described as early as 1992 by Pelosi et al. [12] who performed a laparoscopic appendectomy, and by Navarra et al. [13] who performed a laparoscopic cholecystectomy in 1997. Because SILS can be performed using refinements of existing techniques and technology, it has spread widely to many conventional laparoscopic fields, such as colectomy [14, 15], hysterectomy [16], gastrectomy [17, 18], and the urological field [19, 20].

The SILS technique does not rely on triangulation, which is one of the core principles of conventional laparoscopic surgery, allowing adequate operative exposure while maintaining an ergonomic position for the surgeon and assistant. Consequently, an inherent technical challenge that arises from the SILS technique is that of a compromised view and locomotive field [21]. A small additional port or reduced number of ports (reduced port surgery, RPS) are modifications that may overcome these problems [7, 22]. Our dual-port method provides an additional 12-mm port for the surgeon’s right hand. This port should prevent interference between camera maneuvers and other surgical instruments. A **s**urgical nylon ligature with a straight needle also contributes to maintaining the visual field by lifting up the stomach without any additional surgical instruments. In particular, this procedure does not require any specific expensive instruments other than the SILS port. In combination with the SILS port, the additional 12-mm port and surgical nylon ligature enable the performance of dual-port surgery, even for complex operations such as LAG.

**Fig. 6:**
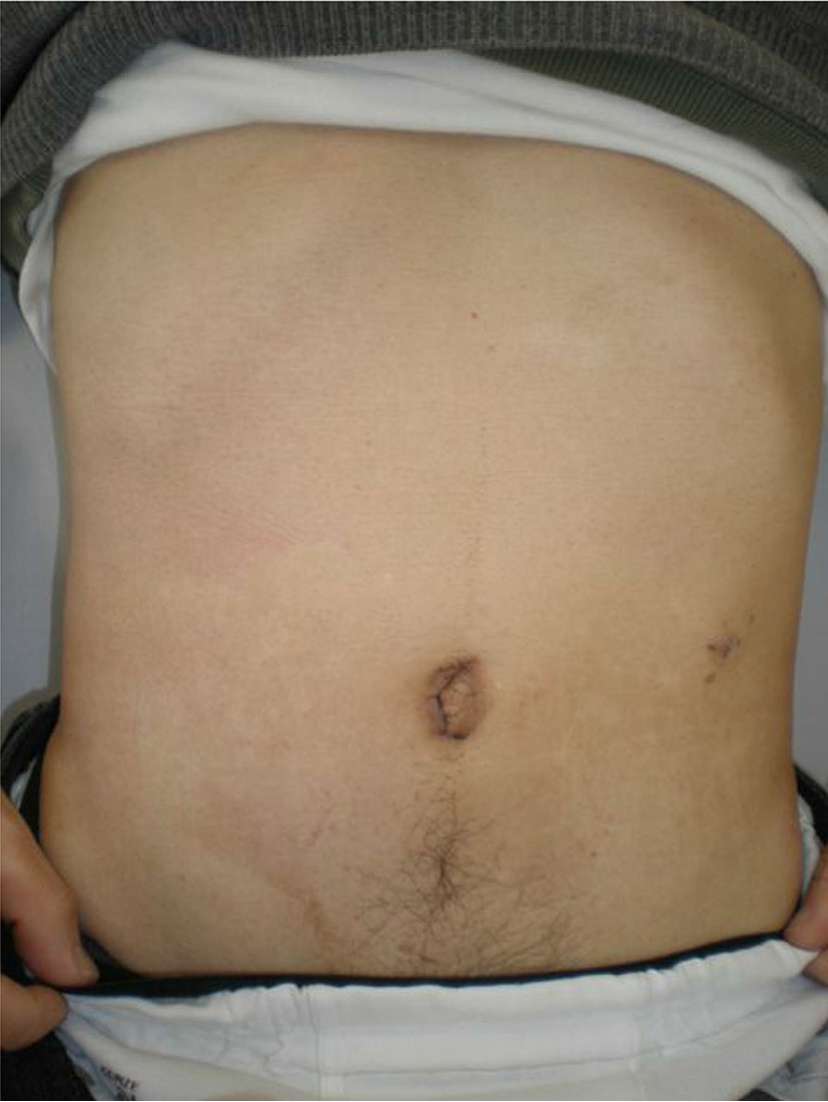
Abdominal incisions after 4 weeks’ recovery

Although the benefits of SILS or RPS over conventional laparoscopic surgery have not been established, some advantages are expected. The cosmetic benefit is a clear advantage of the use of fewer ports such as in SILS or RPS (Fig. 6). Less postoperative pain may be an advantage because of the reduced number of incisions [9], although our data do not show reduced use of analgesics. The reduced invasiveness of SILS and RPS is also notable. In this study, shorter hospital stays and reduced numbers of complications were seen in the RPDG group. In addition, the trend of serum CRP values after surgery was lower for the RPDG group than the conventional LAG group (Fig. 5). CRP is a serum protein, the levels of which rise in response to acute or chronic inflammation. Acute injuries, such as trauma, infection or surgery, cause the release of interleukin-6 and other cytokines that trigger the synthesis of CRP by the liver [23]. During the acute phase response, the level of CRP rapidly increases within 2 h of the acute insult, reaching a peak after around 48 h. Recent research suggests that patients with elevated basal levels of CRP are at increased risk of diabetes, hypertension, and cardiovascular disease [24–26]. Our cases, except for one RPLDG patient (2.23 mg/dl), had normal CRP levels pre-operatively and these values increased gradually until post-operative day 3. Interestingly, the peak level at day 3 showed a clearer difference between the groups than the levels at day**s** 1 and 7, although a statistically significant difference was not seen (*p* = 0.053). The CRP value returned to low levels at post-operative day 7. These acute phase responses suggest that RPDG may be less invasive than conventional LAG. Although it is very difficult to explain why RPDG was less invasive, we have three suggestions. The first is simply “Reduced ports means reduced invasiveness”. Normally, conventional LAG requires four or five ports, occasionally with an additional small incision into the patient’s abdomen so that the total length of the wound size exceeds 5 cm. However, the wound size with our method is less than 4 cm. Although the difference in the total length of the surgical incision is small, the difference in the total area or volume of the abdominal injury is somewhat greater. Therefore, a smaller wound size may be less invasive and reduce subsequent cytokine production from injury sites in the abdominal cavity. Secondly, the positioning of the ports may be associated with a lower complication rate after surgery. Conventional LAG normally requires a few ports or small incisions in the upper abdomen. These upper abdominal injuries may induce minor respiratory problems, such as some degree of alveolar atelectasis, and consequent loss of lung volume, occurring in the same manner as in upper abdominal open surgery and leading to postoperative pain, diminished cough, hypoventilation, and an unfavorable closing volume/functional residual capacity relationship [27]. In particular, 40 % of our patients were over 75 years old; therefore, minor respiratory problems such as post-operative pneumonia or alveolar atelectasis were of concern. The final factor is the mental status after surgery. SILS and RPS are expected to have a better outcome in terms of the mental status of the patients after surgery because of less abdominal injury, including the cosmetic benefit. This patient satisfaction may contribute to early walking and easy breathing after surgery, resulting in lower complication rates and the lower serum CRP levels observed in the RPDG group.

In addition, port-related complications such as organ damage, adhesion, bleeding, wound infection, and hernias may be less frequent in SILS and RPS because of the lower number of ports. Larger studies of RCT are needed to confirm these advantages in the future.
